# Supplementary material for: A turquoise fluorescence lifetime-based biosensor for quantitative imaging of intracellular calcium
Source: Nat Commun. 2021 Dec 9;12:7159. doi: 10.1038/s41467-021-27249-w (PMC8660884; doi:10.1038/s41467-021-27249-w)
Supplement: Supplementary file 1 — Supplementary information [file 41467_2021_27249_MOESM1_ESM.pdf]

A turquoise fluorescence lifetime-based biosensor for quantitative imaging of intracellular calcium

Franka H. van der Linden[1], Eike K. Mahlandt[1], Janine J.G. Arts[1,2], Joep Beumer[3], Jens Puschhof[3], Saskia M.A. de Man[1], Anna O. Chertkova[1], Bas Ponsioen[4], Hans Clevers[3], Jaap D. van Buul[1,2], Marten Postma[1], Theodorus W.J. Gadella Jr.[1] & Joachim Goedhart[1,*]

Affiliations:
[1]Swammerdam Institute for Life Sciences, Section of Molecular Cytology, van Leeuwenhoek Centre for Advanced Microscopy, University of Amsterdam, Amsterdam, the Netherlands.

[2] Department of Molecular Hematology at Sanquin Research and Landsteiner Laboratory, Academic Medical Centre, University of Amsterdam, the Netherlands

[3]Oncode Institute, Hubrecht Institute, Royal Netherlands Academy of Arts and Sciences and University Medical Center, Utrecht, the Netherlands.

[4]Oncode Institute, Center for Molecular Medicine, University Medical Centre Utrecht, Utrecht, the Netherlands.

[*]Correspondence: j.goedhart@uva.nl

Supplement

Supplemental Notes 1 - 4

Supplemental Figures 1 - 15

Supplemental Tables 1 - 5

Supplemental Notes

Supplementary note 1. Construction of the dual expression plasmid pFHL.

To allow fast and easy screening of sensor candidates, a dedicated expression vector was designed, termed pFHL, inspired by the plasmids pDuEx (pDress[1] were the mTurquoise2, large spatial linker and P2A sequences were removed from the plasmid using NheI restriction sites) and pTorPE[2]. The protein of interest, in this case a candidate sensor, is under control of a CMV promotor for mammalian expression and a rhamnose promotor for bacterial expression. This will eliminate the need of transferring the candidate sensor to a vector for mammalian expression after bacterial screening. At the N-terminus, the sensor is fused to a TorA tag, a 6xHis-tag and an Xpress-tag. The TorA tag primes transport of the sensor to the periplasm of bacteria[3]. As a result, changing the outer environment, for example adding a compound to an agar plate or to a liquid culture, will directly influence the candidate sensor. Also, easy periplasmic isolation will yield a relatively clean protein, ready for quick testing[2]. The 6xHis-tag and the Xpress-tag can be used for protein isolation.

The optimal concentration of rhamnose for expression in *E. coli* using the pFHL vector was determined to be 0.4% (w/v) (**Supplementary Figure S3A**). The performance of the dual expression vector was verified in *E. coli* and HeLa cells, and compared to the pTorPE plasmid. When expressed in *E. coli*, the R-GECO1 sensor reacted to changing calcium concentration in liquid growth medium (**Supplementary Figure S3B**). A bigger response was obtained when the sensor was first isolated by isolation of the periplasmid fluid with an osmotic shock. The contrast was lower using the pFHL vector compared to the TorPE vector. However, we found that the contrast was sufficient for screening. The vector also allowed expression in HeLa cells, where addition of ionomycin and extra calcium resulted in a robust intracellular calcium increase, necessary for lifetime measurements (**Figure 2B**).

Supplementary note 2. Influence of residue 150 in mTurquoise2 on fluorescence lifetime.

Amino acid V150 is positioned close to the chromophore in mTurquoise2 and therefore we suspected it to affect the fluorescent lifetime of the protein. We randomly mutated this position. Fluorescent and dark colonies were picked and collected on two plates, from which the modulation lifetime was measured using frequency domain FLIM (**Supplementary Figure S5**) using a custom build FLIM setup and analysis as described before[4]. Lifetimes between 2.4-4.0 ns were recorded among the fluorescent colonies.

Supplementary note 3. Changing the calcium concentration in the periplasm of bacteria on agar plates.

E. coli cells expressing Tq-Ca-FLITS.0 were grown on LB-agar plates. The modulation lifetime (τ_M) of the sensor was measured before and > 5 min after 1, 2 or 3 stimulations with a droplet of calcium or chelator EDTA **(Supplementary Figure S6)**. The lifetime was recorded of the whole plate using a custom build FLIM setup and analysis as described before[4].

Addition of calcium increased the τ_M from 2.70±0.03 ns to 2.83±0.02 ns (mean±sd). Adding more calcium to a colony did not further increase the lifetime. Addition of 200 mM EDTA decreased the lifetime to 2.51±0.07 ns, 2.33±0.08 ns and 2.15±0.11 ns for 1, 2 and 3 drops respectively. The results show that the sensors in bacteria on LB-agar are primarily in the high lifetime state and that the sensors can indeed be influenced from the outer environment, as a result of expression in the periplasmic space.

Supplementary note 4. Calcium concentrations during transendothelial migration

Several reports have documented the use of calcium sensitive probes to study changes in calcium levels in endothelial cells upon their interaction with white blood cells. These studies are summarized in **Supplementary Table S4**.

The increase in calcium concentration that is observed in endothelial cells varies. This can be partially attributed to the different experimental conditions that are used. Here, we have tried to approach the physiological situation as close as possible by (i) studying TEM under flow at 37 °C, (ii) pretreating the endothelial monolayer with TNF to mimic inflamed conditions, (iii) activating the freshly isolated leukocytes by 20 min incubation at 37 °C and (iv) omitting any of the perturbations that are necessary for labeling cells with fluorescent dyes. Specifically, the use of a genetically encoded probe does not require pre-incubation at room temperature, does not need helper reagents (Pluronic) and omits issues with dye leakage and incomplete hydrolysis. Moreover, Tq-Ca-FLITS uses visible light for excitation (in contrast to Indo-1 and Fura-2 which require UV) and enables direct, intensity-independent quantification.

Finally, we have fluorescently labeled both cell types and can therefore precisely analyze the interaction between two cell types and distinguish the different phases of TEM.

		EC (M-1 cm-1)	QY	Kd (nM)	pKa	Intrinsic brightness	Ref.
**GCaMP3**	*apo*	11000	0.2	542	8.73	2200	a
	sat	50000	0.44		6.6	22000	
**GCaMP6f**	*apo*	2761	0.57	297	7.72	1574	b
	sat	66293	0.66		6.25	43753	
**GCaMP6s**	*apo*	2118	0.41	147	7.54	868	b
	sat	70117	0.64		6.03	44875	
**jGCaMP7f**	*apo*	2358	0.47	174	7.88	1108	b
	sat	56028	0.59		6.5	33057	
**jGCaMP7s**	*apo*	554	0.58	68	7.69	321	b
	sat	53068	0.65		6.36	34494	
**jGCaMP7c**	*apo*	1541	0.5	298	8.66	771	b
	sat	49566	0.59		6.66	29244	
**jGCaMP7b**	*apo*	5668	0.59	82	7.82	3344	b
	sat	56462	0.6		6.37	33877	
**EGFP**		55000	0.6		5.9	33000	c
**K-GECO1**	*apo*	19000	0.12	165	6.71, 8.23	2280	d
	sat	61000	0.45		6.34	27450	
**FusionRed**		94500	0.19		4.6	17955	e
**R-GECO1**	*apo*	15000	0.06	482	8.9	900	a
	sat	51000	0.2		6.59	10200	
**mApple**		82000	0.47		6.5	38540	f
**RCaMP1h**	*apo*	20800	0.13	1127	7.1	2704	g
	sat	63600	0.6		5.7	38160	
**jRCaMP1b**	*apo*	25300	0.15	712	6.4	3795	g
	sat	53400	0.54		5.5	28836	
**mRuby**		112000	0.35		4.4	39200	h
**mScarlet**		100000	0.7		5.3	70000	f
**Tq-Ca-FLITS**	*apo*	30600	0.25	360	4.35	7650	This study
	sat	33700	0.75		4.71, 5.91	25275	
**mTurquoise2**		30000	0.93		3.67	27900	i,j

Supplementary Figure S1. Properties of current intensity-based calcium sensors. Properties of the parent FP are also included: EGFP for all green sensors, FusionRed for K-GECO1, mApple for R-GECO1, mRuby for RCaMP1h and jRCaMP1b, mTurquoise2 for Tq-Ca-FLITS. mScarlet demonstrates the theoretical possibility for improvement of the brightness of the red sensors. Horizontal color bars indicate the relative intrinsic brightness compared to sensors and FPs of the same color (green, red or cyan). Tq-Ca-FLITS published here was added for comparison, which has a notably much higher relative intrinsic brightness in the calcium free state compared to other sensors.

EC indicates the extinction coefficient, QY the quantum yield.

References: a – Zhao et al. (2011)[2], b – Dana et al. (2019)[5], c – Patterson et al. (2001)[6], d – Shen et al. (2018)[7], e – Shemiakina et al. (2012)[8], f – Bindels et al. (2017)[9], g – Dana et al. (2016)[10], h – Kredel et al. (2009)[11], i – Goedhart et al. (2012)[12], j – Cranfill et al. (2016)[13].

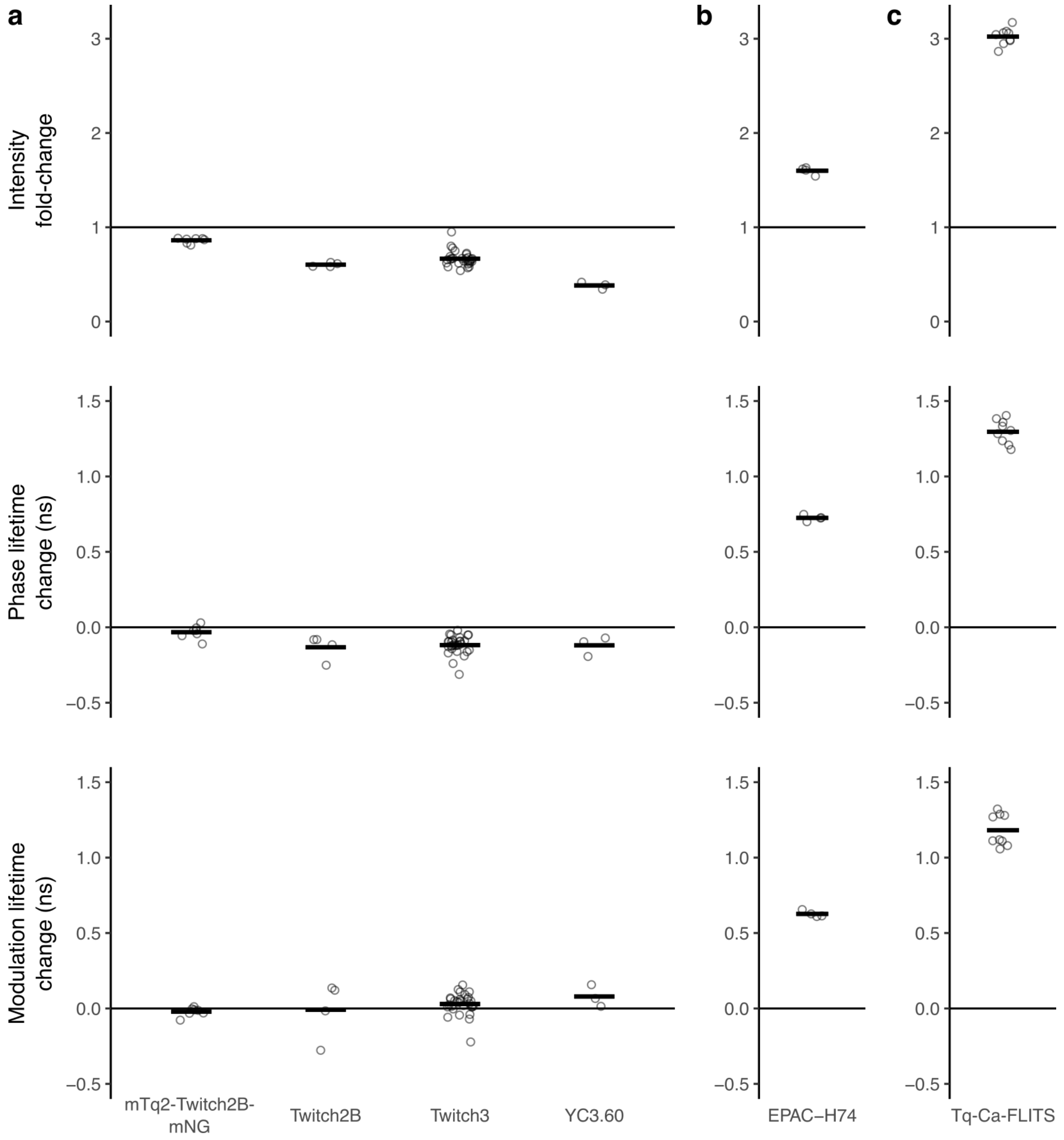

Supplementary Figure S2. Intensity and lifetime contrast of biosensors. A) YCaM3.60, Twitch3, Twitch2B and mTq2-Twitch2B-mNG were transiently expressed in HeLa cells. Intensity and donor lifetimes were recorded before and after stimulation with 14 mM ionomycin combined with 5 mM $CaCl_2$ (*n*=3 to 29). The intensity fold-change (top panel) and the absolute modulation and phase lifetime change (middle and bottom panel) of the donor fluorescent protein are plotted. **B)** Published donor intensity and lifetime change of EPAC-H74**,** a FRET-FLIM sensor for cyclic AMP[14] (*n*=4) with a substantial lifetime contrast. **C)** For comparison we show the performance of new Turquoise calcium biosensor (Tq-Ca-FLITS). HeLa cells were transiently transfected with the sensor and stimulated with 14 mM ionomycin and 5 mM $CaCl_2$ while imaging (*n*=9). Individual cell responses (circles) and their mean (line) are plotted for all sensors. Source data are provided as a Source Data file.

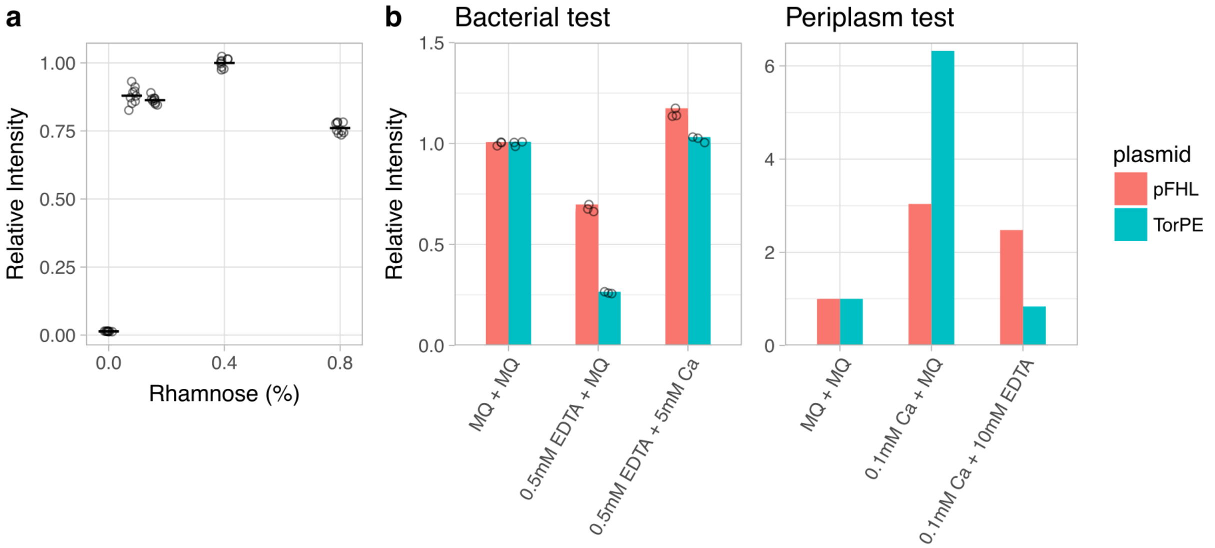

Supplementary Figure S3. Verification of the new dual expression plasmid named pFHL. A) Normalized fluorescence intensities are shown of overnight grown *E. coli* cultures expressing R-GECO1 at different rhamnose concentrations, using the pFHL vector that contains a rhamnose promotor. Separate measurements (circles, *n*=9) and the mean are indicated. **B)** R-GECO1 responds to changing calcium concentrations in *E. coli* (Bacterial test, *n*=3) and in isolated periplasmic fluid (Periplasm test, *n*=1), by sequential addition of calcium and chelator EDTA. MilliQ water (MQ) was used a control. The performance of the pFHL vector in this test was compared to the pTorPE vector. Source data are provided as a Source Data file.

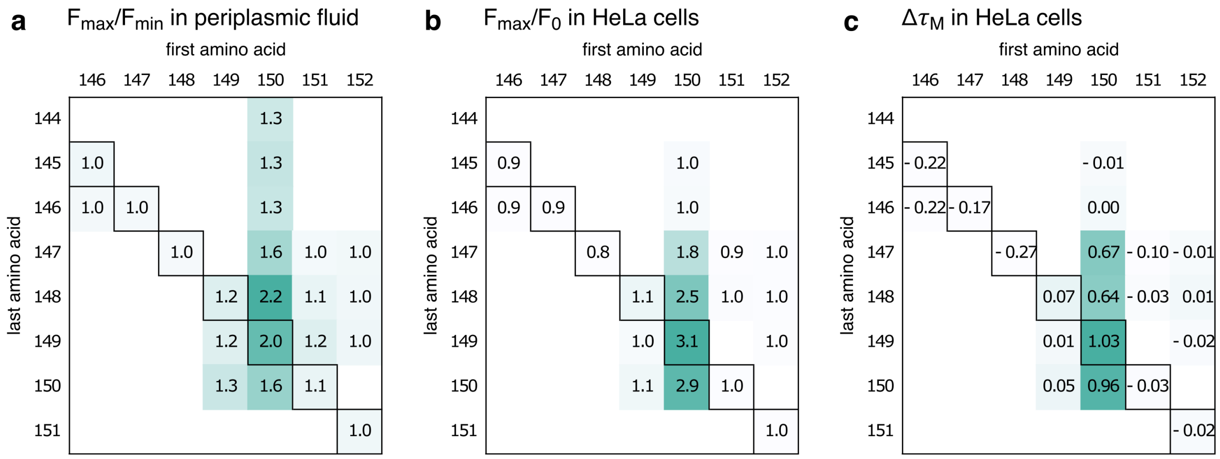

Supplementary Figure S4. Screening the insertion site and the number of indels for the creation of Tq-Ca-FLITS. A) Intensity fold-change of different sensor variants in periplasmic shock fluid isolated from *E. coli*. **B)** Intensity fold-change and **C)** absolute modulation lifetime change (ns) in HeLa cells upon stimulation with ionomycin and calcium. In all panels are the first amino acid of mTurquoise2 after the M13 peptide and the last amino acid before the CaM indicated. See also the design of Tq-Ca-FLITS in **Figure 2A.** On the diagonal axis (black boxes) are the sensor variants that were created to find the ideal position to insert the CaM and the M13 peptide (mean of 8 to 23 cells). The other variants contain 2 to 5 indels around the insertion site (mean of 2 to 8 cells). Source data are provided as a Source Data file.

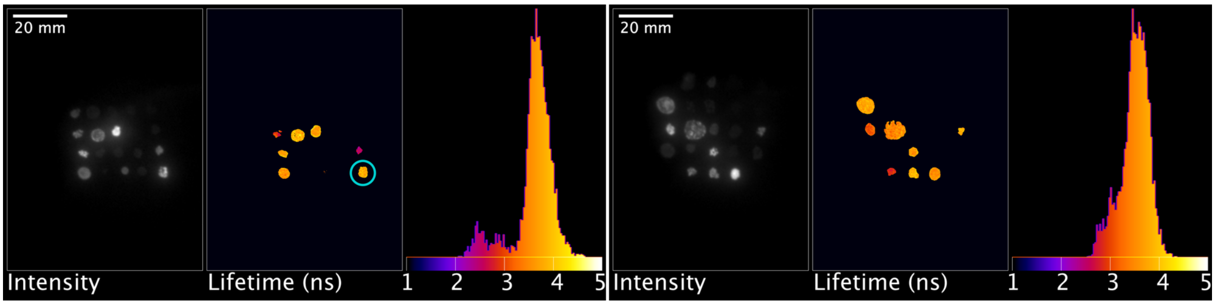

Supplementary Figure S5. Lifetimes displayed by mTurquoise2 mutants in *E. coli* on agar plates. Intensity and modulation lifetime of mTurquoise2 mutated at position V150 are displayed. Two agar plates were imaged. A histogram of the modulation lifetimes is shown for each plate. A circle indicates non-mutated mTurquoise2. The panel was generated by an imageJ macro[1].

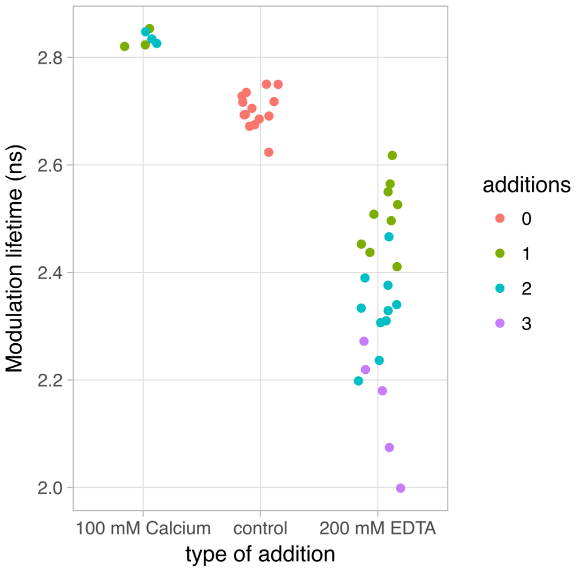

Supplementary Figure S6. Modulation lifetime of bacterial colonies expressing Tq-Ca-FLITS.0. Colonies were stimulated 1, 2 or 3 times with a droplet of 100 mM calcium or 200 mM EDTA. Calcium increases the modulation lifetime and each drop of EDTA decreases the lifetime. Circles represent the modulation lifetime of individual colonies (*n*=3 to *n*=14). Source data are provided as a Source Data file.

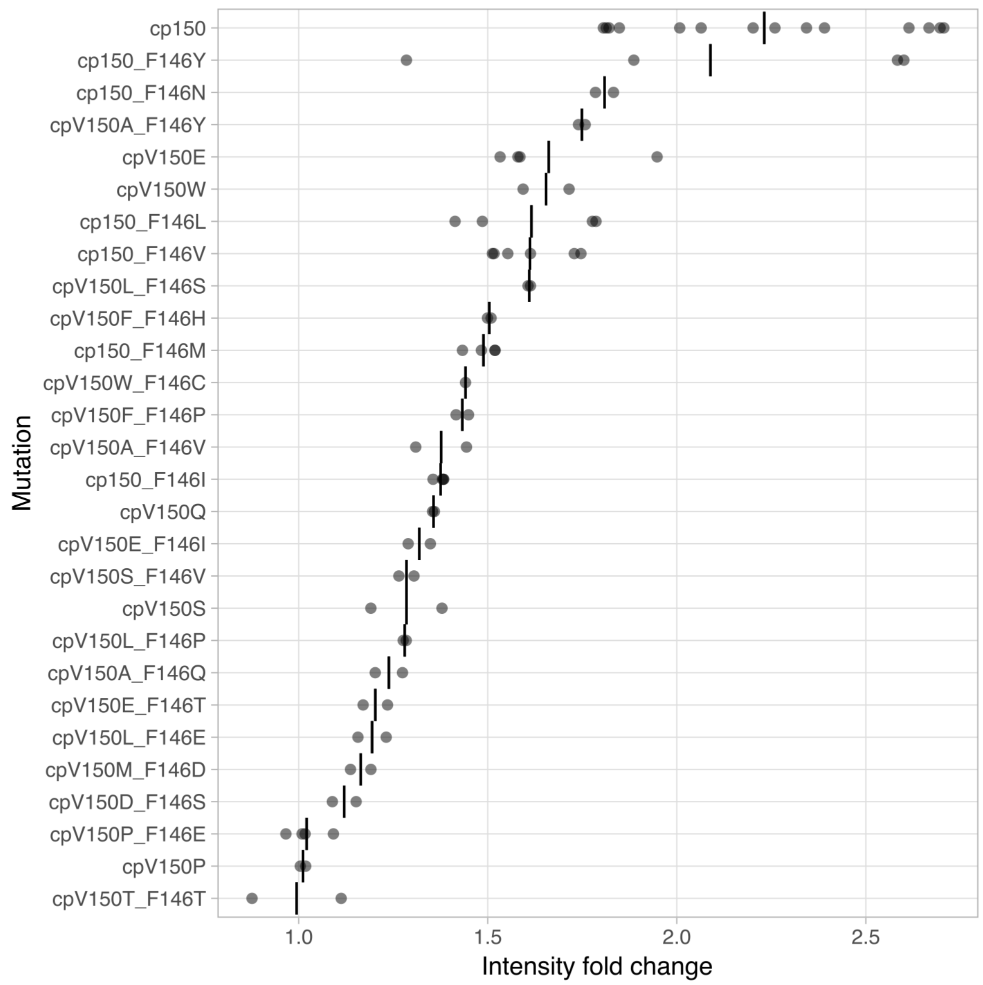

Supplementary Figure S7. Intensity fold-change (F_{max}/F_0) of mutant sensors as measured by the periplasmic test. Gray circles indicate individual experiments (*n*=2 to *n*=14) and black lines indicate the mean per mutant. The original amino acids at position 150 and 146 are V and F. Source data are provided as a Source Data file.

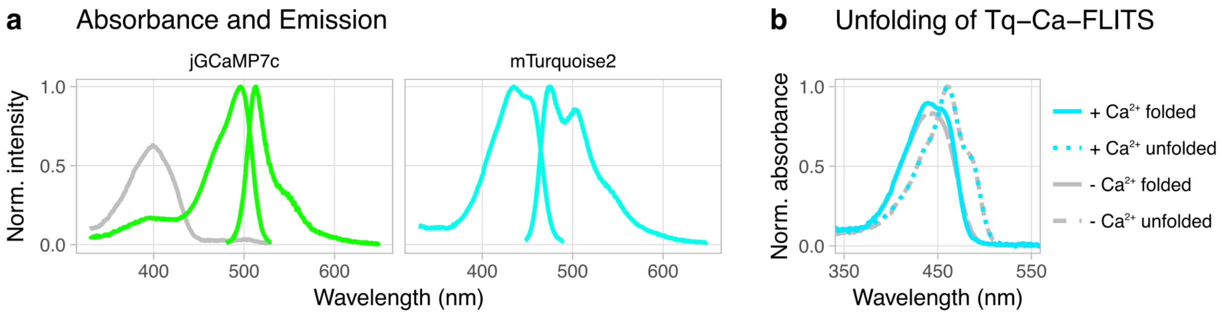

Supplementary Figure S8. Spectra of Tq-Ca-FLITS and jGCaMP7c *in vitro*. A) Absorption and excitation spectra of: jGCaMP7c (left panel) in the calcium bound (green) and unbound state (gray) and mTurquoise2 (right panel). **B)** Change in absorbance spectra of Tq-Ca-FLITS by unfolding the calcium bound (blue) and unbound (gray) state with 1 M NaOH. Source data are provided as a Source Data file.

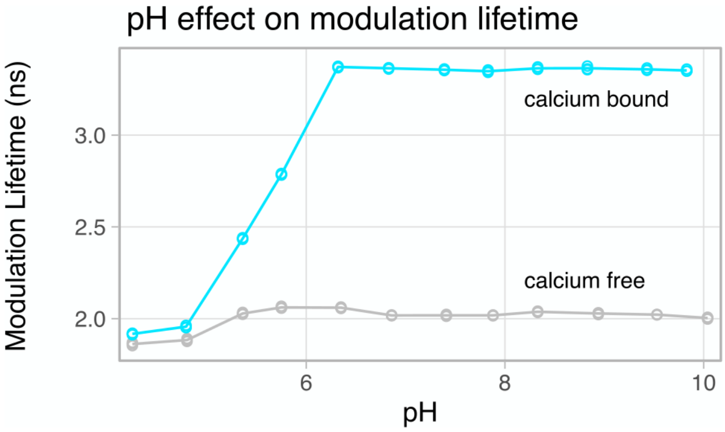

Supplementary Figure S9. The modulation lifetime of Tq-Ca-FLITS *in vitro* in presence and absence of calcium. The modulation lifetime is stable above pH 6.2 (*n*=3 indicated by individual circles, with line average). Source data are provided as a Source Data file.

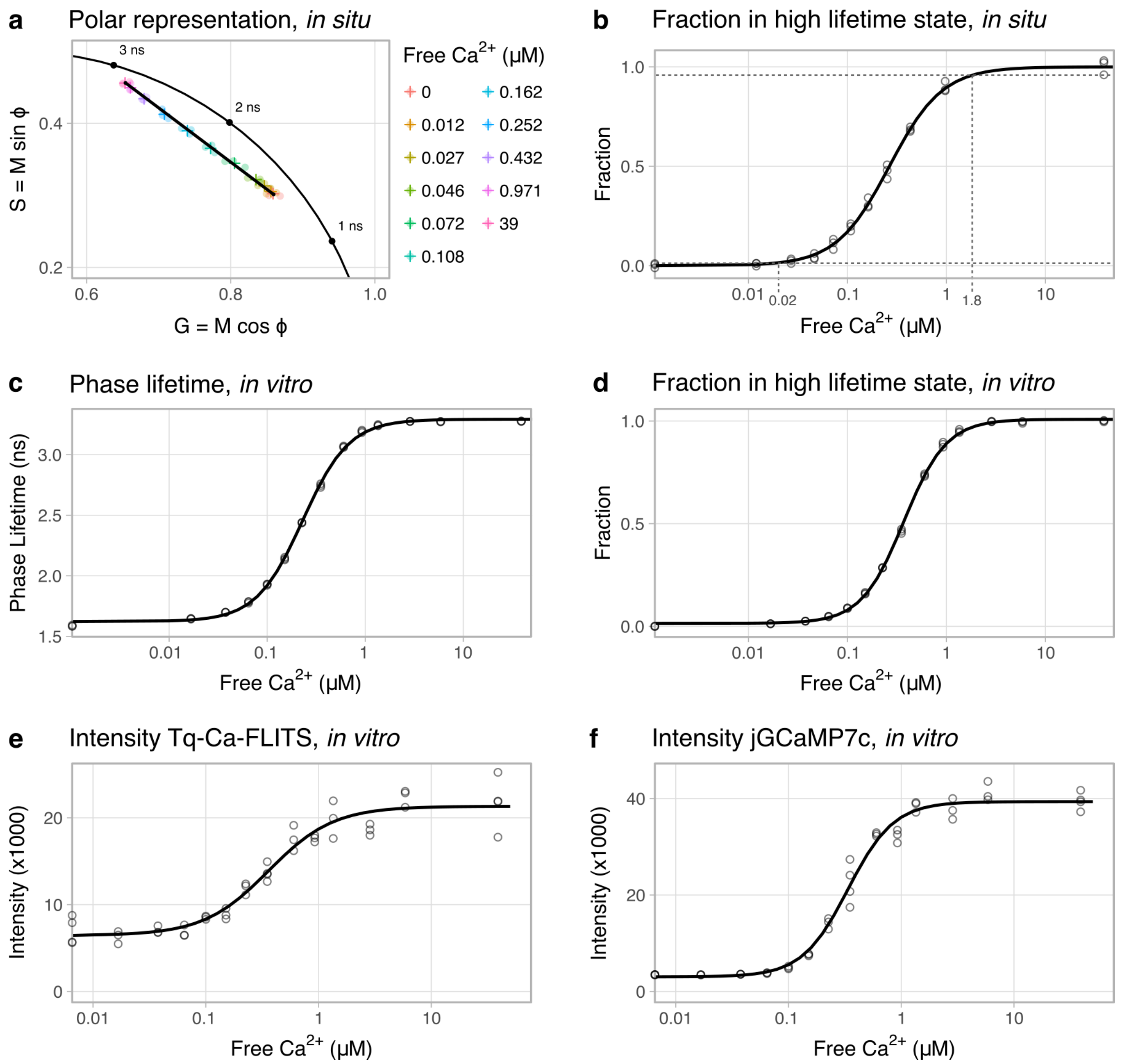

Supplementary Figure S10. Calcium sensitivity of Tq-Ca-FLITS and jGCaMP7c. A) *In situ* calibration of Tq-Ca-FLITS represented on a polar plot (*n*=3), where '*M*' and '*Φ*' relate to the modulation and phase lifetime, respectively. Each dot represents the average of all pixels in a view with > 20 cells. The black line runs from the position of the calcium free state to the position of the calcium bound state. The fraction of sensor in the high state for each concentration was calculated from a projection on the black line, corrected for the intensity contribution of the two states. **B)** *In situ* calibration of the fraction of Tq-Ca-FLITS in the high state as determined from a polar plot in panel A (n=3). The dotted horizontal lines indicate the borders of the measurable range, based on the 95% confidence interval of the fraction of the lowest and highest calcium concentrations. **C)** *In vitro* calibration of the phase lifetime of Ta-Ca-FLITS (*n*=3). **D)** *In vitro* calibration of the fraction of Tq-Ca-FLITS in the high state as determined from a polar plot (n=3). **E and F)** Sensitivity of the intensity readout of Tq-Ca-FLITS and jGCaMP7c *in vitro* (each *n*=3). In panels **B-F** the circles indicate individual measurements, and the line represents the fitted model. In all figures the calcium concentration is plotted on a logarithmic scale. Source data are provided as a Source Data file.

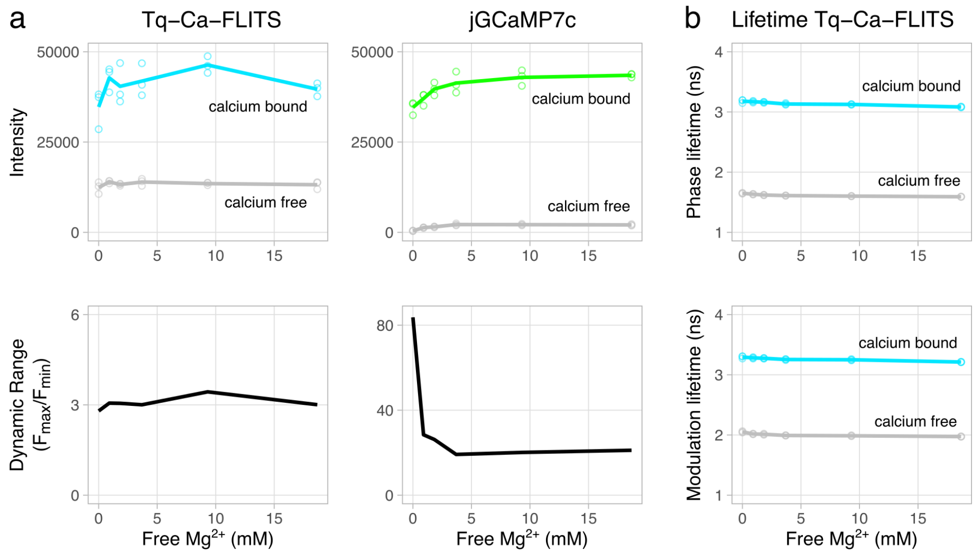

Supplementary Figure S11. Magnesium sensitivity of Tq-Ca-FLITS and jGCaMP7c in vitro. A) Fluorescence intensity for the calcium bound (blue/green) and free (gray) state (top panels), and the corresponding dynamic range (bottom panels). **B)** Phase and modulation lifetime of Tq-Ca-FLITS in response to magnesium, for both the calcium bound (blue) and free (gray) state. Individual measurements (n=3) are indicated by dots, the line represents the average. Source data are provided as a Source Data file.

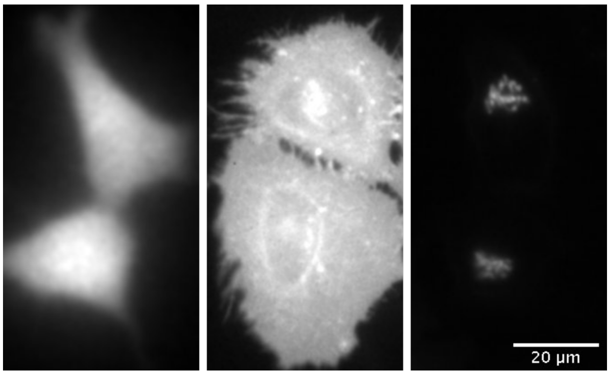

Supplementary Figure S12. Organelle targeting of Tq-Ca-FLITS. The probe localizes to the cytoplasm without a targeting sequence (left panel), to the plasma membrane using an Lck-tag (middle panel), and the Golgi using a giantin-tag (right panel). All panels show two cells, representative of at least 15 cells.

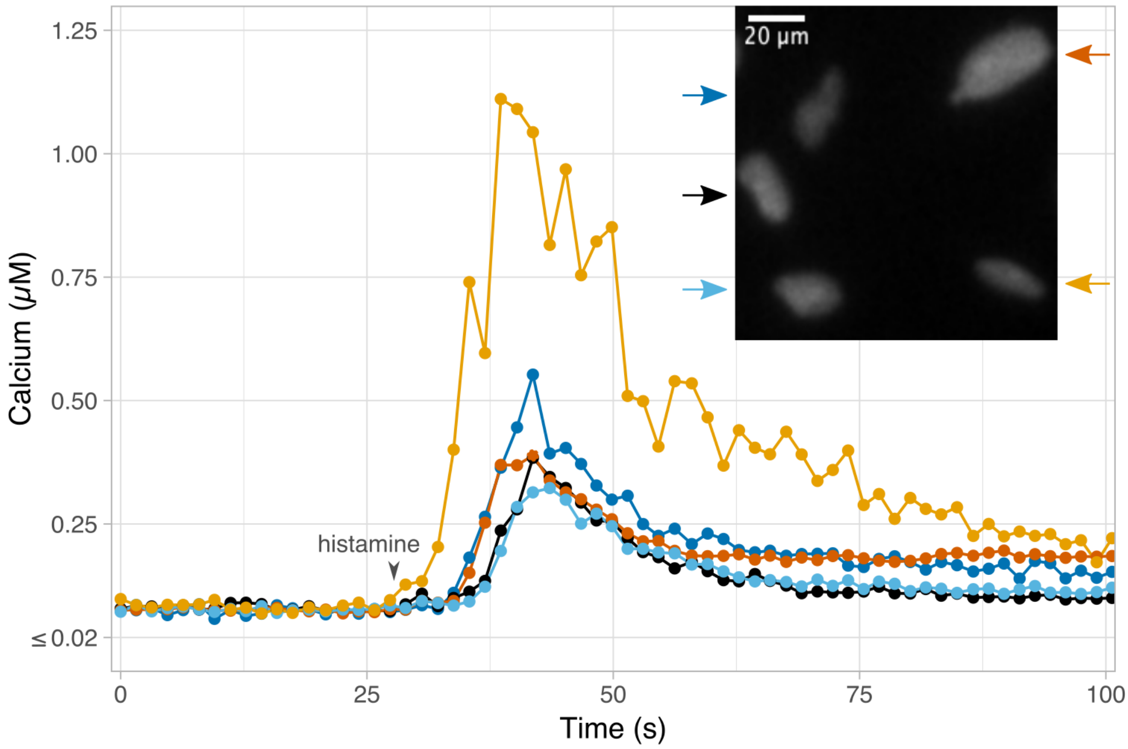

Supplementary Figure S13. Temporal increase in calcium in nuclei of HeLa cells upon stimulation with histamine. Tq-Ca-FLITS was targeted in the nucleus by a 3xNLS-tag. Responses of five representative cells are shown (*n*=95), indicated in the inset by arrows of the same color. The moment of addition of 2 µM histamine is indicated in the graph. Source data are provided as a Source Data file.

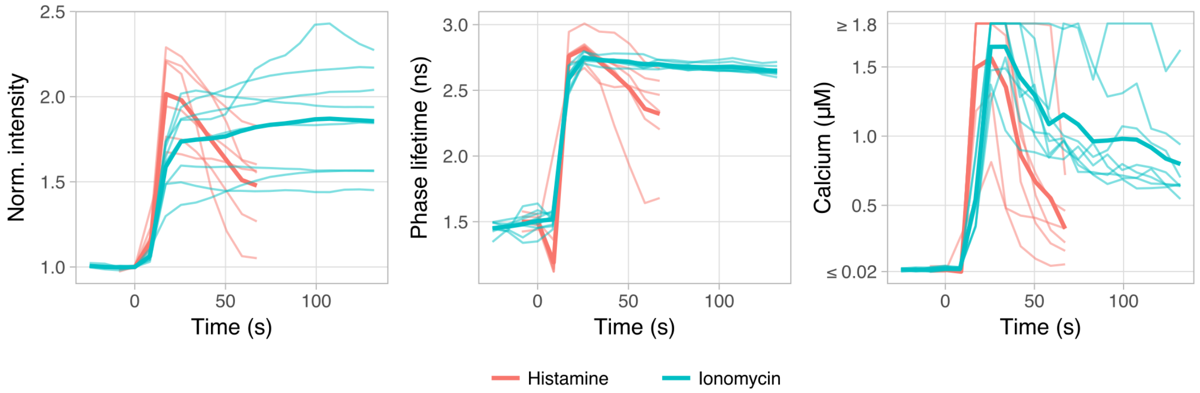

Supplementary Figure S14. ECs expressing Tq-Ca-FLITS respond to histamine or ionomycin after the TEM assay. The change in intensity (left panel), change in lifetime (middle panel) and corresponding calcium concentration (right panel) are shown for individual cells with thin lines. Thick lines represent the mean. Tq-Ca-FLITS is fully functional in all measured cells (histamine *n*=6, ionomycin *n*=8). Source data are provided as a Source Data file.

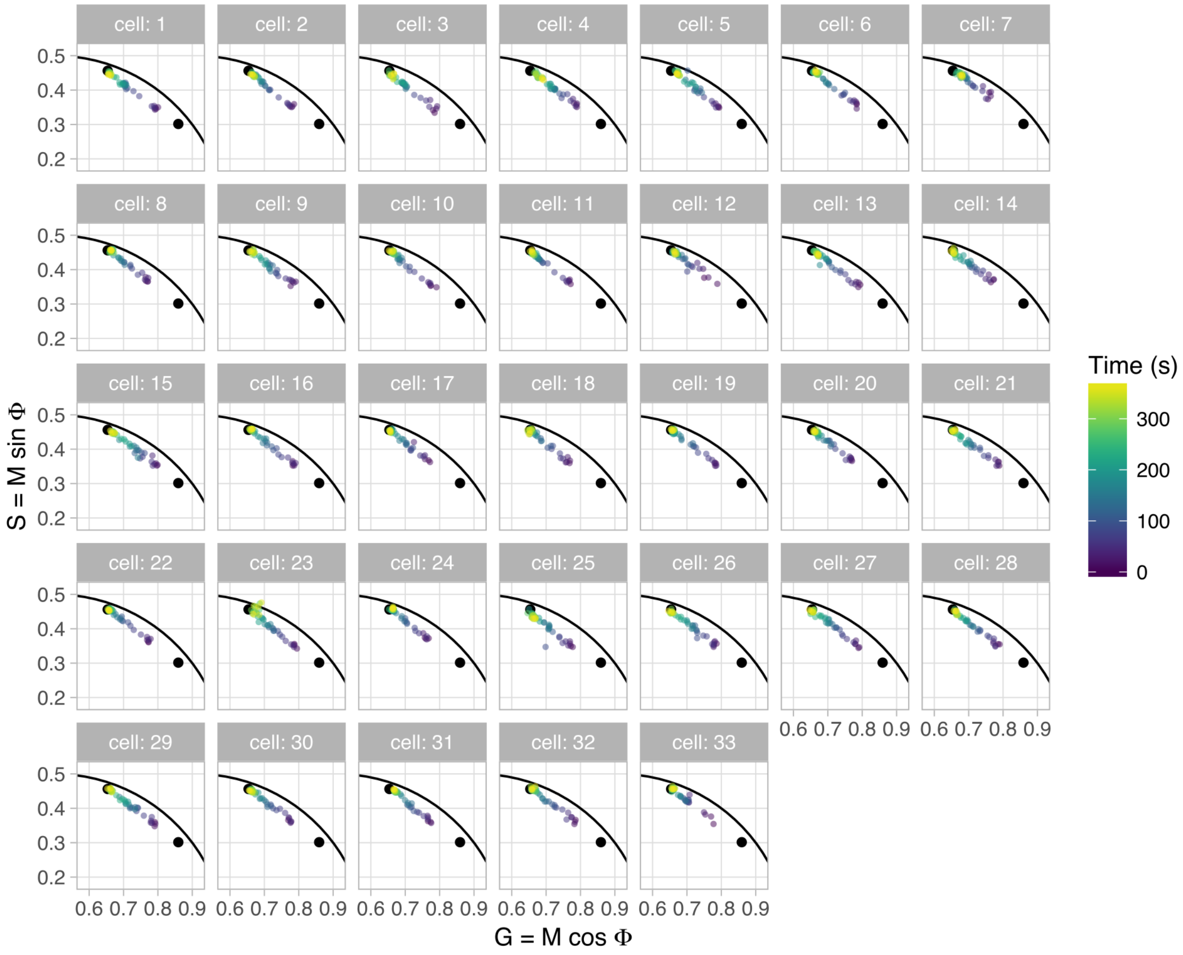

Supplementary Figure S15. Tq-Ca-FLITS in human small intestinal organoid reacts to addition of 0.25 % (w/v) Triton-X100 and 12.5 mM calcium. A polar plot representation of the change in lifetime of individual cells (n=33) is given by the colored dots. The black dots indicate the positions of the calcium bound (upper left) and calcium free (lower right) states of the sensor. '*M*' and '*Φ*' relate to the modulation and phase lifetime, respectively. At the start of the measurement all cells show a lifetime near the calcium free state and towards the end of the measurement move completely (with a few exceptions) to the lifetime of the calcium bound state. Source data are provided as a Source Data file.

Supplementary Table S1. Variation of the output of different calcium sensors in HeLa cells.

Sensor	Measured property	state	N	mean	sd	CV
**Tq-Ca-FLITS**	phase lifetime (ns)	pre	110	1.45	0.036	0.025
		post	110	2.77	0.035	0.013
	modulation lifetime (ns)	pre	110	1.84	0.055	0.030
		post	110	2.94	0.034	0.012
**jRCaMP1b**	phase lifetime (ns)	pre	29	1.60	0.120	0.075
		post	56	2.78	0.040	0.014
	modulation lifetime (ns)	pre	29	2.35	0.103	0.044
		post	56	3.02	0.055	0.018
**RCaMP1h**	phase lifetime (ns)	pre	31	1.05	0.049	0.047
		post	69	2.74	0.040	0.014
	modulation lifetime (ns)	pre	31	1.72	0.079	0.046
		post	69	2.97	0.049	0.017
**MatryoshCaMP6s**	intensity ratio (GFP/OFP)	pre	17	0.64	0.312	0.486
		post		3.18	0.813	0.255
**YCaM3.60**	FRET ratio (YFP/CFP)	pre	11	1.68	0.271	0.161
		post		5.24	0.564	0.108

The respective fluorescent property was measured for each sensor before (pre) and after (post) addition of 14 mM ionomycin combined with 5 mM $CaCl_2$. For lifetime measurements both the phase and modulation lifetimes are indicated. The mean, standard deviation (sd) and coefficient of variation (CV, sd divided by mean) of a number of individually measured cells (N) are given. CV is a number for the variation, independent of the absolute value, and can therefore be used to compare the variation of different types of readouts. Data for MatryoshCaMP6s and YCaM3.60 was acquired with microscope 1 (see **Figure 1**). Tq-Ca-FLITS published here was added for comparison and shows the lowest CV of all sensors. Source data are provided as a Source Data file.

Supplementary Table S2. Properties of Tq-Ca-FLITS compared to jGCaMP7c and mTurquoise2

		Spectral properties						Sensor properties		
		λ_{abs} (nm)	λ_{ex} (nm)	λ_{em} (nm)	QY	ε (M^{-1} cm^{-1})	pK_a [n]	K_d [n] {CI} (nM) pH 7.2	F_{max}/F_{min} *in vitro* pH 7.0	F_{max}/F_0 *in situ*
Tq-Ca-FLITS	Apo	442	446	489	0.25	30600 [440]	4.35 [0.86]	360 [1.51] {289-455}	3.51	3.02
	Sat	439	446	481	0.75	33700 [440]	1: 4.71 [0.70]<br>2: 5.91 [3.58]			
jGCaMP7c	Apo	399	n.d.	n.d.	0.50$	1541**	9.11 [0.60]	337 [2.14] {309-369}	127.8	n.d.
	Sat	496	500	513	0.59$	49566**	6.82 [1.06]			
mTurquoise2		435	437	475	0.93*	30000* [434]	3.67 [1.55]	n.a.	n.a.	n.a.

Spectral properties: λ_{abs} – Absorbance maximum. λ_{ex} – Excitation maximum. λ_{em} – Emission maximum. QY – Quantum Yield relative to mTurquoise2. ε – Extinction coefficient, determined at the indicated wavelength. pK_a [n] – Apparant pK_a values, with Hill coefficient [n]. A model with two pK_a values was used for the calcium saturated state of Tq-Ca-FLITS.
Sensor properites: K_d [n] – Apparent K_d, with the Hill coefficient [n] and the 95% confidence interval {CI} indicated. F_{max}/F_{min} – ratio of maximum over minimum fluorescence intensity, or calcium bound state over calcium unbound state *in vitro*. F_{max}/F_0 – maximum intensity over starting intensity *in situ*, as determined by stimulation with ionomycin and calcium (n=3).
n.a./n.d.: not applicable/not determined.
* Determined earlier in our lab[12]
** Published by others[5]

Supplementary Table S3. Lifetime properties of Tq-Ca-FLITS for quantitative measurements

	In vitro calibration			*In situ* calibration		
	τ_φ {CI} (ns)	τ_M {CI} (ns)	K_d [n] {CI} (nM)	τ_φ {CI} (ns)	τ_M {CI} (ns)	K_d [n] {CI} (nM)
Apo	1.62 {1.61-1.64}	1.97 {1.96-1.98}	372 [2.03] {364-380}	1.40 {1.37-1.42}	1.80 {1.75-1.86}	265 [1.63] {257-274}
Sat	3.29 {3.28-3.30}	3.35 {3.34-3.36}		2.78 {2.74-2.81}	3.01 {2.96-3.07}	

τ_φ – phase lifetime. τ_M – Modulation lifetime. K_d [n] – Apparent K_d, with the Hill coefficient [n], determined from the position on a polar plot. 95% confidence intervals {CI} are indicated.

If you have made it this far & you like our work, please mention @joachimgoedhart in a tweet

Supplementary Table S4. Summary of studies that investigate calcium during TEM.

Calcium increase	Endothe-lial cells	White blood cell	Probe (loading conditions)	Single cell	Flow	Stage	Ref.
> 800 nM	HUVEC	PMN (10:1) +fMLP	Fura-2 (18 °C & Pluronic)	yes	no	Adhesion	[15]
Yes, qualitative	HAEC	PMN (5-10:1)	Indo-1 (23 °C, RT)	no	no	Adhesion	[16]
Yes, qualitative	HUVEC	CD65+/NK (20:1 ratio)	Fluo-3 (22 °C & Pluronic)	yes	no	Adhesion	[17]
180 nM	Lung HMVEC	Lymphocyte (10^5 /ml)	Fura-2 (37 °C & Pluronic)	no	no	Adhesion	[18]
Qualitative: ~60% of cells	HMEC-1	Monocytes (10:1)	Indo-1 (RT)	yes	no	Adhesion	[19]
~200 nM	HAEC	Monocytes	Fura-2 (24 °C)	yes	no	Adhesion	[20]
Qualitative: ~25% of cells	HUVEC	Neutrophils	YCaM	yes	yes	Rolling	[21]
Qualitative: None of the cells	HUVEC	Neutrophils	YCaM	yes	yes	Crawling	[21]
No: < 80 nM	HUVEC	Neutrophils	Tq-Ca-FLITS	yes	yes	Crawling & diapedesis	This study

Supplementary Table S5. Primers for construction of Tq-Ca-FLITS

No.	Sequence	Use
	Creation pFHL plasmid, template is indicated.	
1	TCGGGGAAATGTGCGCGG	C1 plasmid as template
2	TCAGGGGATAACGCAGGAAAG	C1 plasmid as template
3	tcacatgttctttcctgcgttatcccctgaGGCCGGCCTATTAATAGTAATC	pDuEx plasmid as template
4	cctctttaaacccatGGTATATCTCCTTCTTATAGTTAAACAAAATTATTTC	pDuEx plasmid as template
5	agaaggagatataccATGGGTTTAAAGAGGAGAAAG	TorPE-R-GECO1 as template
6	cctgaggttaatcacAAGCTTCTACTTCGCTGTC	TorPE-R-GECO1 as template
7	gcgaagtagaagcttGTGATTAACCTCAGGTGCAG	pDuEx plasmid as template
8	caaataggggttccgcgcacatttccccgaGCATGCTTTCAGCAAAAAC	pDuEx plasmid as template
	Sensors with variation in insertion site	
9	gctgagctcacccgtggttTTTAGCGACAACGTCTATATC	FW cp146 sensor
10	gtcacgcgtGTAGTTGTACTCCAGCTTG	RV cp146 sensor
11	gctgagctcacccgtggttAGCGACAACGTCTATATC	FW cp147 sensor
12	gtcacgcgtAAAGTAGTTGTACTCCAGC	RV cp147 sensor
13	gctgagctcacccgtggttGACAACGTCTATATCACCG	FW cp148 sensor
14	gtcacgcgtGCTAAAGTAGTTGTACTCC	RV cp148 sensor
15	gctgagctcacccgtggttAACGTCTATATCACCGCC	FW cp149 sensor
16	gtcacgcgtGTCGCTAAAGTAGTTGTAC	RV cp149 sensor
17	gctgagctcacccgtggttGTCTATATCACCGCCGAC	FW cp150 sensor
18	gtcacgcgtGTTGTCGCTAAAGTAGTTG	RV cp150 sensor
19	gctgagctcacccgtggttTATATCACCGCCGACAAGC	FW cp151 sensor
20	gtcacgcgtGACGTTGTCGCTAAAGTAG	RV cp151 sensor
21	gctgagctcacccgtggttATCACCGCCGACAAGCAG	FW cp152 sensor
22	gtcacgcgtATAGACGTTGTCGCTAAAG	RV cp152 sensor
23	gctgagctcacccgtggttACCCAGTCCAAGCTGAGC	FW cp203 sensor
24	gtcacgcgtGCTCAGGTAGTGGTTGTC	RV cp203 sensor
25	gctgagctcacccgtggttCAGTCCAAGCTGAGCAAAG	FW cp204 sensor
26	gtcacgcgtGGTGCTCAGGTAGTGGTTG	RV cp204 sensor
27	gtcacgcgtGTTGTACTCCAGCTTGTGC	RV cp145 sensor
	Mutations	
28	gctgagctcacccgtggtt**NNK**TATATCACCGCCGACAAG	V150X on Tq-Ca-FLITS.0
29	gtcacgcgtGTTGTCGCT**MNN**GTAGTTGTACTCCAGC	F146X on Tq-Ca-FLITS.0
30	gctgagctcacccgtggtt**GCG**TATATCACCGCCGACAAG	V150A on Tq-Ca-FLITS.0
31	gctgagctcacccgtggtt**MTC**TATATCACCGCCGACAAG	V150I/L on Tq-Ca-FLITS.0
32	gtcacgcgtGTTGTCGCT**ATA**GTAGTTG	F146Y on Tq-Ca-FLITS.0
33	CTACTTTAGCGACAAC**NNK**TATATCACCGCCGAC	V150X on mTurquoise2
34	GTCGGCGGTGATATA**MNN**GTTGTCGCTAAAGTAG	V150X on mTurquoise2
	Organelle targeting	
35	gctaccggtcgccaccATGGTCGACTCTTCACG	FW, all variants
36	ttttgtacacCTTCGCTGTCATCATTTGGACAAACTC	RV, Golgi and membrane
37	ttttgtacacCTACTTCGCTGTCATCATTTGGACAAACTC	RV, including STOP codon, nuclear and cytoplasmic
	Sequencing	
38	GTGGACAGCAAATGGGTCG	FW, sensor variants
39	CCCAGAGACCGCATCACC	RV, sensor variants

Annealing regions are indicated by capital letters, mutations in bold.

Supplementary References

1. Bindels, D. S., Postma, M., Haarbosch, L., van Weeren, L. & Gadella, T. W. J. Multiparameter screening method for developing optimized red-fluorescent proteins. *Nat. Protoc.* **15**, 450–478 (2020).
2. Zhao, Y. *et al.* An Expanded Palette of Genetically Encoded Ca2+ indicators. *Science (80-.).* **333**, 1888–1891 (2011).
3. Barrett, C. M. L., Ray, N., Thomas, J. D., Robinson, C. & Bolhuis, A. Quantitative export of a reporter protein, GFP, by the twin-arginine translocation pathway in Escherichia coli. *Biochem. Biophys. Res. Commun.* **304**, 279–284 (2003).
4. Bindels, D. S. *et al. Chapter 16: Optimization of Fluorescent Proteins*. *Fluorescence Spectroscopy and Microscopy* **1076**, (Methods in Molecular Biology, 2014).
5. Dana, H. *et al.* High-performance calcium sensors for imaging activity in neuronal populations and microcompartments. *Nat. Methods* **16**, 649–657 (2019).
6. Patterson, G., Day, R. N. & Piston, D. Fluorescent protein spectra. *J. Cell Sci.* **114**, 837–838 (2001).
7. Shen, Y. *et al.* A genetically encoded Ca2+indicator based on circularly permutated sea anemone red fluorescent protein eqFP578. *BMC Biol.* **16**, 1–16 (2018).
8. Shemiakina, I. I. *et al.* A monomeric red fluorescent protein with low cytotoxicity. *Nat. Commun.* **3**, (2012).
9. Mastop, M. *et al.* Characterization of a spectrally diverse set of fluorescent proteins as FRET acceptors for mTurquoise2. *Sci. Rep.* **7**, 1–18 (2017).
10. Dana, H. *et al.* Sensitive red protein calcium indicators for imaging neural activity. *Elife* **5**, 1–24 (2016).
11. Kredel, S. *et al.* mRuby, a bright monomeric red fluorescent protein for labeling of subcellular structures. *PLoS One* **4**, (2009).
12. Goedhart, J. *et al.* Structure-guided evolution of cyan fluorescent proteins towards a quantum yield of 93%. *Nat. Commun.* **3**, (2012).
13. Cranfill, P. J. *et al.* Quantitative assessment of fluorescent proteins. *Nat. Methods* **13**, 557–562 (2016).
14. Klarenbeek, J. B., Goedhart, J., Hink, M. A., Gadella, T. W. J. & Jalink, K. A mTurquoise-based cAMP sensor for both FLIM and ratiometric read-out has improved dynamic range. *PLoS One* **6**, 2–7 (2011).
15. Huang, A. J. *et al.* Endothelial cell cytosolic free calcium regulates neutrophil migration across monolayers of endothelial cells. *J. Cell Biol.* **120**, 1371–1380 (1993).
16. Ziegelstein, R. C. *et al.* Initial contact and subsequent adhesion of human neutrophils or monocytes to human aortic endothelial cells releases an endothelial intracellular calcium store. *Circulation* **90**, 1899–1907 (1994).
17. Pfau, S. *et al.* Lymphocyte adhesion-dependent calcium signaling in human endothelial cells. *J. Cell Biol.* **128**, 969–978 (1995).
18. Ehringer, W. D., Edwards, M. J., Wintergerst, K. A., Cox, A. & Miller, F. N. An Increase in Endothelial Intracellular Calcium and F-Actin Precedes the Extravasation of Interleukin-2-Activated Lymphocytes. *Microcirculation* **5**, 71–80 (1998).
19. Kielbassa-Schnepp, K. *et al.* Endothelial intracellular Ca2+ release following monocyte adhesion is required for the transendothelial migration of monocytes. *Cell Calcium* **30**, 29–40 (2001).
20. Peterson, M. D. *et al.* Monocyte-induced endothelial calcium signaling mediates early xenogeneic endothelial activation. *Am. J. Transplant.* **5**, 237–247 (2005).
21. Heemskerk, N., Asimuddin, M., Oort, C., van Rijssel, J. & van Buul, J. D. Annexin A2 Limits Neutrophil Transendothelial Migration by Organizing the Spatial Distribution of ICAM-1. *J. Immunol.* **196**, 2767–2778 (2016).
