# Supplementary material for: A turquoise fluorescence lifetime-based biosensor for quantitative imaging of intracellular calcium
Source: Nat Commun. 2021 Dec 9;12:7159. doi: 10.1038/s41467-021-27249-w (PMC8660884; doi:10.1038/s41467-021-27249-w)
Supplement: Supplementary file 2 — Reporting Summary [file 41467_2021_27249_MOESM2_ESM.pdf]

nature research

Corresponding author(s): J.Goedhart

Last updated by author(s): Sep 22, 2021

Reporting Summary

Nature Research wishes to improve the reproducibility of the work that we publish. This form provides structure for consistency and transparency in reporting. For further information on Nature Research policies, see our Editorial Policies and the Editorial Policy Checklist.

Statistics

For all statistical analyses, confirm that the following items are present in the figure legend, table legend, main text, or Methods section.

n/a	Confirmed	
☐	☒	The exact sample size (*n*) for each experimental group/condition, given as a discrete number and unit of measurement
☐	☒	A statement on whether measurements were taken from distinct samples or whether the same sample was measured repeatedly
☒	☐	The statistical test(s) used AND whether they are one- or two-sided<br>*Only common tests should be described solely by name; describe more complex techniques in the Methods section.*
☒	☐	A description of all covariates tested
☒	☐	A description of any assumptions or corrections, such as tests of normality and adjustment for multiple comparisons
☐	☒	A full description of the statistical parameters including central tendency (e.g. means) or other basic estimates (e.g. regression coefficient) AND variation (e.g. standard deviation) or associated estimates of uncertainty (e.g. confidence intervals)
☒	☐	For null hypothesis testing, the test statistic (e.g. *F*, *t*, *r*) with confidence intervals, effect sizes, degrees of freedom and *P* value noted<br>*Give P values as exact values whenever suitable.*
☒	☐	For Bayesian analysis, information on the choice of priors and Markov chain Monte Carlo settings
☒	☐	For hierarchical and complex designs, identification of the appropriate level for tests and full reporting of outcomes
☒	☐	Estimates of effect sizes (e.g. Cohen's *d*, Pearson's *r*), indicating how they were calculated

Our web collection on statistics for biologists contains articles on many of the points above.

Software and code

Policy information about availability of computer code

Data collection	Emission and absorbance spectra were measured using the FL WinLab software (Perkin Elmer).<br>Fluorescence of samples in a 96-wells plate was recorded with the KC4™ software (Bio-Tek).<br>Lifetime images were acquired using either the LI-FLIM software (version 1.2.13) when using a Nikon/Lamberts Instruments setup or an in-house developed MATLAB (v6.1) script described previously (Merzlyak, E.M. et al. Nat. Methods 4, 555-557 (2007)) when using a home-build Zeiss FLIM setup.
Data analysis	R Studio (version 1.0.136) was used for fitting of the calcium calibration and pH sensitivity curves, using the R Stats Package (version 3.3.3).<br>FACS data was analysed with FlowJo (version 10.4.2).<br>Lifetime images were processed either with an in house-developed MATLAB script or an ImageJ script.<br>ImageJ version 1.52k was used for further analysis of microscopy images to convert lifetime data into concentration images.<br>Custom code and scripts are available through GitHub: https://github.com/Franka-van-der-Linden/Quantitative-Calcium-Imaging

For manuscripts utilizing custom algorithms or software that are central to the research but not yet described in published literature, software must be made available to editors and reviewers. We strongly encourage code deposition in a community repository (e.g. GitHub). See the Nature Research guidelines for submitting code & software for further information.

Data

Policy information about availability of data

All manuscripts must include a data availability statement. This statement should provide the following information, where applicable:

- Accession codes, unique identifiers, or web links for publicly available datasets
- A list of figures that have associated raw data
- A description of any restrictions on data availability

The data produced in this study study are available within the article and its Supplementary Information.
All raw data will be available at Zenodo.org upon publication.
Plasmids are deposited for distribution through Addgene (www.addgene.org). The plasmids and corresponding addgene numbers are: pFHL-Tq-Ca-FLITS: #129628, 3xnls-Tq-Ca-FLITS: #129626, Lck-Tq-Ca-FLITS: #129627, pPB-3xnls-Tq-Ca-FLITS: #145030, pLV-H2B-Maroon-P2A-3xnls-Tq-Ca-FLITS: #145027.

Field-specific reporting

Please select the one below that is the best fit for your research. If you are not sure, read the appropriate sections before making your selection.

- [x] Life sciences
- [ ] Behavioural & social sciences
- [ ] Ecological, evolutionary & environmental sciences

For a reference copy of the document with all sections, see nature.com/documents/nr-reporting-summary-flat.pdf

Life sciences study design

All studies must disclose on these points even when the disclosure is negative.

Sample size	No a priori sample-size calculation was performed. For experiments with primary cells, the sample size was defined by sample availability. A minimum of three independent samples was measured whenever possible. This is typically the standard for the experiments performs so that a reasonable range of variability between samples can be accounted for.
Data exclusions	No data was excluded from the analysis
Replication	All attempts of replication showed a consistent pattern and were deemed successful. Multiple independent experiments were conducted as reported to ensure reproducibility of the experimental findings were validated. Number of replicates is indicated in the manuscript for each experiment.
Randomization	Not applicable
Blinding	Not applicable

Reporting for specific materials, systems and methods

We require information from authors about some types of materials, experimental systems and methods used in many studies. Here, indicate whether each material, system or method listed is relevant to your study. If you are not sure if a list item applies to your research, read the appropriate section before selecting a response.

Materials & experimental systems

n/a	Involved in the study	
x		Antibodies
	x	Eukaryotic cell lines
x		Palaeontology and archaeology
x		Animals and other organisms
x		Human research participants
x		Clinical data
x		Dual use research of concern

Methods

n/a	Involved in the study	
x		ChIP-seq
x		Flow cytometry
x		MRI-based neuroimaging

Eukaryotic cell lines

Policy information about cell lines

Cell line source(s)	Primary Human Umbilical Vein Endothelial Cells were acquired from Lonza (P1052, Cat #C2519A).<br>Whole blood from healthy donors was acquired from Sanquin, for isolation of polymorphonuclear cells.<br>HeLa cells lines were obtained from ATCC (CCL-2).<br>The N39 ileum cell line that was used to generate the organoids was from a patient sample from non-transformed, normal mucosa was taken for this study. The study was approved by the UMC Utrecht (Utrecht, The Netherlands) ethical committee and was in accordance with the Declaration of Helsinki and according to Dutch law. This study is compliant with all relevant ethical regulations regarding research involving human participants.
Authentication	No authentication was performed
Mycoplasma contamination	The HeLa and N39 cell line tested negative for mycoplasm. PCR tests are routinely performed approximately every 6 months
Commonly misidentified lines<br>(See ICLAC register)	No commonly misidentified cell lines were used in this study.
