# Supplementary material for: A turquoise fluorescence lifetime-based biosensor for quantitative imaging of intracellular calcium
Source: Nat Commun. 2021 Dec 9;12:7159. doi: 10.1038/s41467-021-27249-w (PMC8660884; doi:10.1038/s41467-021-27249-w)
Supplement: Supplementary file 4 — Description of Additional Supplementary Files [file 41467_2021_27249_MOESM4_ESM.pdf]

Title: Supplementary Movie 1:
Description: Calcium calibration in HeLa cells that express a nuclear targeted Tq-Ca-FLITS biosensor. The intensity and phase lifetime data are shown after cell permeabilization and equilibration at different external calcium concentrations.

Title: Supplementary Movie 2:
Description: Calcium concentrations in HeLa cells that express a nuclear targeted Tq-Ca-FLITS biosensor before and after stimulation with 2 µM histamine. Displayed are on the left the intensity of the probe and on the right the calcium concentration calculated from the lifetime data in false color according to the color scale.

Title: Supplementary Movie 3:
Description: Calcium levels in endothelial cells monitored with plasma membrane targeted Tq-Ca-FLITS before and after stimulation with 1 µM histamine. Displayed are on the left the intensity of the probe and on the right the calcium concentration calculated from the lifetime data in false color according to the color scale.

Title: Supplementary Movie 4:
Description: Calcium levels in endothelial cells monitored with plasma membrane targeted Tq-Ca-FLITS during transendothelial migration. Displayed are on the left the intensities of the probe and the neutrophil (in cyan and red respectively) and on the right the calcium concentration calculated from the lifetime data in false color according to the color scale. The white region of interest shows the location of the neutrophil, showing that calcium is not elevated in this region.

Title: Supplementary Movie 5:
Description: Calcium changes measured in nuclei of human small intestinal organoids stimulated with 10 µg/ml GPBAR-A at 25 sec. Displayed are on the left the intensity of the probe and on the right the calcium concentration calculated from the lifetime data in false color according to the color scale.
